# The potential of HEART score to detect the severity of coronary artery disease according to SYNTAX score

**DOI:** 10.1038/s41598-023-34213-9

**Published:** 2023-05-04

**Authors:** Amirhossein Salimi, Abdolali Zolghadrasli, Soodeh Jahangiri, Mohammad Reza Hatamnejad, Mehdi Bazrafshan, Peyman Izadpanah, Fatemeh Dehghani, Amir Askarinejad, Maryam Salimi, Hamed Bazrafshan Drissi

**Affiliations:** 1 Student Research Committee, Shahid Sadoughi University of Medical Sciences, Yazd, Iran; 2 Cardiovascular Research Center, Shiraz University of Medical Sciences, Shiraz, Iran; 3 Student Research Committee, Shiraz University of Medical Sciences, Shiraz, Iran; 4 Rajaie Cardiovascular Medical and Research Center, Iran university of medical sciences, Tehran, Iran

**Keywords:** Cardiology, Medical research

## Abstract

Clinical scoring systems such as the HEART score can predict major adverse cardiovascular events, but they cannot be used to demonstrate the degree and severity of coronary artery disease. We investigated the potential of HEART Score in detecting the existence and severity of coronary artery disease based on SYNTAX score. This multi-centric cross-sectional study investigated patients referred to the cardiac emergency departments of three hospitals between January 2018 and January 2020. Data including age, gender, risk factors, comorbidities, 12-lead ECG, blood pressure and echocardiogram were recorded for all the participants. Serum troponin I level was measured on admission and 6 h later. Coronary angiography was done via the femoral or radial route. HEART and SYNTAX scores were calculated for all patients and their association was assessed. 300 patients (65% female) with mean age of 58.42 ± 12.42 years were included. mean HEART Score was 5.76 ± 1.56 (min = 3, max = 9), and mean SYNTAX score was 14.82 ± 11.42 (min = 0, max = 44.5). Pearson correlation coefficient was 0.493 between HEART Score and SYNTAX score which was statistically significant (P < 0.001). We found that HEART Score of more than 6 is 52% sensitive and 74.7% specific to detect extensive coronary artery involvement (SNTAX score ≥ 23). The present study showed that the HEART score has a moderate and positive correlation with the SYNTAX score and HEART score with a cut-off value of 6 is a predictor for SYNTAX score of ≥ 23.

## Introduction

While chest pain (CP) is a major cause of referral to the emergency departments (ED), only less than 25% of these patients actually have acute coronary syndrome (ACS). Patients with CP are usually hospitalized and undergo further testing and even invasive procedures like coronary angiography which leads to unnecessary hospitalization and cost^1,2^. On the other hand, normal levels of troponin or normal electrocardiograms (ECGs) do not necessarily exclude the presence of ACS^3^. Therefore, the ability to reduce hospital costs and its burden on the public health system relies on the ability to correctly risk stratify patients which will also directly influence management plan^4^.

Scoring systems such as SYNTAX and Gensini scores are basically scoring systems used to determine the extent and the severity of coronary artery disease and to determine treatment choice based on angiographic data^5^ and these scoring systems have been used in some studies to determine the prognosis of patients and have been observed to have successful results. Scoring systems such as Heart score and Grace score are scoring systems that try to show the prognosis of patients according to their clinical status^6,7^ and the main expectation from these scoring systems is to be an indicator of coronary plaque burden and extent rather than determining the severity of coronary artery disease.

HEART score (HS) is valuable in risk stratification of patients presenting with chest pain^8^. It is based on five elements including history, ECG, age, coronary risk factors, and troponin level^9^. Patients with a risk score of three or less are considered low-risk while a score more than seven is considered high risk and those between these two values are regarded as moderate risk for major adverse cardiac events (MACE)^10^.

On the other hand, the SYNTAX score (SS) has been developed to measure the complexity of coronary artery disease (CAD) and so determine the best revascularization strategy for patients with CAD. High SS indicates that patients are more prone to major MACE and need early intervention. Although SS is a well-validated method but requires coronary angiogram which is an invasive diagnostic intervention^11–15^. Therefore, an alternative non-invasive clinical scoring system such as the HS to determine the extent of the underlying CAD would be clinically relevant. However, previous studies on HS mainly sought to find the incidence of MACE, and the correlation of HS with angiographic findings have never been investigated^16–19^.

In this study, we aim to assess the ability of the HS to predict the extension of coronary artery disease according to SS in patients with CP.

## Materials and methods

### Study design and participant selection

This multi-centric cross-sectional study was conducted for a period of 2 years from January 2018 to January 2020. This research was approved by the ethics committee of Shiraz University of Medical Sciences. Written informed consent was obtained from all patients after explaining the purpose of the study. We made sure that the participation of patients did not influence diagnostic or therapeutic approaches.

All patients who were referred to the cardiac EDs of the three main hospitals affiliated with Shiraz University of Medical Sciences were included.

Patients with non-coronary chest pain including aortic syndrome, pulmonary embolism, pneumothorax, and pneumonia, and those who were not candidates for coronary angiography were excluded. Furthermore, patients who had ST-segment elevation on electrocardiogram (ECG) were immediately transferred to the catheterization unit and consequently omitted from the study.

### Data acquisition, HEART score, and SYNTAX score

A pre-defined questionnaire was filled out to obtain the patients' demographic data including age, gender, and risk factors such as smoking, hypertension (HTN), diabetes mellitus (DM) and hyperlipidemia as well as clinical data including blood pressure.

A 12-lead ECG was obtained from all the participants upon entry to the ED. An experienced cardiologist who was blinded to the study interpreted ECGs^20^. A copy of the admission ECG was recorded in each patient’s data file.

Echocardiography was performed by an expert cardiologist with the USA GE HEALTHCARE VIVID7 DIMENSION device.

Serum troponin I level was measured in all the participants on admission and 6 h later via troponin assay ELISA kit. The Troponin value of the first blood sample was used for the HS calculation.

HS and SS were calculated by an independent cardiologist, blinded to the study and patient's characteristics. HS was calculated for each patient using initial admission data^21,22^. Furthermore, patients were divided into three groups; low risk (HS ≤ 3), moderate risk (3 ≤ HS > 7), and high risk (HS ≥ 7) based on HS.

**Figure 1:**
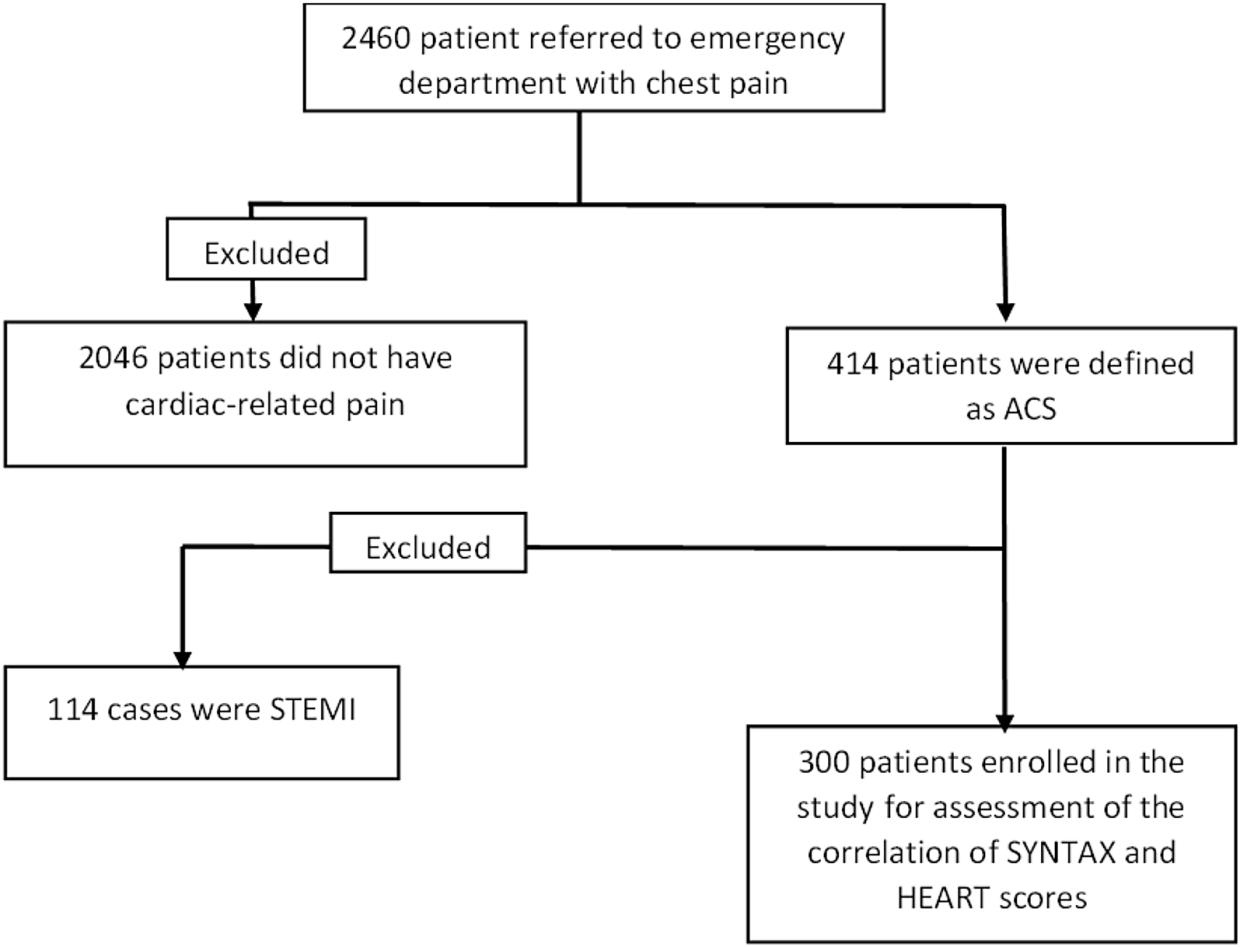
Pathway design of the study.

Coronary angiography was done via the femoral or radial route by an experienced cardiologist. SS was calculated according to the software available online at (The Syntax Score website. https://syntaxscore.org/. Accessed 29 Aug 2022). SS of ≥ 23 was considered as high-risk for occlusive coronary artery disease. The patients were included in the study consecutively (Fig. 1).

### Statistical analysis

Statistical analysis was done using SPSS software, version 23. Categorical and continuous variables were presented as number (%) and mean ± SD, respectively. Statistical significance of scores’ differences within the groups of patients was evaluated by independent samples T-test and One-way ANOVA test. The association of quantitative parameters and scores were examined via Pearson correlation test and presented by r-coefficient and p-value. The relationship between the SS and its potential predictors was analysed by univariate and multivariate logistic regression models and illustrated by odds ratio, 95% confidence interval (CI), and corresponding statistical significance. Receiver operating characteristic (ROC) curves were used to determine the accuracy, sensitivity, and specificity of significant parameters (the result of multi-variate analysis) to predict the SS. P-values less than 0.05 were considered statistically significant.

### Ethics approval

All procedures involving human participants were in accordance with the ethical standards of the institutional and national research committees and with the 1964 Helsinki declaration and its later amendments or comparable ethical standards. Approval was granted by the ethical committee of Shiraz University of Medical Sciences.

### Informed consent

Written Informed consent was obtained from all individual participants included in the study. The purpose of this research was entirely explained to the patients. They were assured that their information would be kept confidential by the researcher.

## Results

As illustrated by the flowchart in Fig. 1, during the investigation period, 2460 patients were referred to the emergency department with complaint of CP, and cardiac-related pain was confirmed in 414 of them. After application of the exclusion criteria, 114 patients were omitted and the remaining 300 patients were included in the study.

The patients’ baseline demographics are described in Table 1. The mean age was 58.42 ± 12.42 years (range 20–87) of which 65% were male and 35% were female. HTN was the main risk factor (60%) in our study’s population; other risk factors were DM (40%), hyperlipidemia (35%), and smoking (25%). According to our analysis, the HS and SS scores were associated with age, the presence of DM and HTN, angiography outcomes and management group of the patients (P-value < 0.05). Even though P. value of < 0.001 has been calculated between all group (SVD, 2VD, 3VD, SF or MB), it was not significant between just the two group of 2VD (6.59 ± 1.50) and 3VD (6.27 ± 1.28) statistically significant (P = 0.1380). For the management group also the P. value of < 0.001 has been calculated between all of them (Medical, PCI-SV, PCI-MV, CABG). It was not significant just between two group of MV PCI and CABG (P = 0.2981) even though the mean of PCI-MV was higher. However, we could not demonstrate a significant association between HS and SS with gender, smoking status and the presence of hyperlipidemia. Moreover, no statistically significant association was found between HS and SS with blood pressure on presentation and ejection fraction on echocardiograms.

**Table 1:** Baseline characteristics of the patients.

Variables	Total patients^a^	HEART score^b^		SYNTAX score^b^	
		Mean ± SD or r	P-value	Mean ± SD or r	P-value
Age	58.42 ± 12.42	0.474	** < 0.001**	0.411	** < 0.001**
< 45	36 (12%)	4.15 ± 1.21	** < 0.001**	6.80 ± 2.33	** < 0.001**
45 ≤ age < 64	159 (53%)	5.45 ± 1.27		13.5 ± 11.1	
≥ 64	105 (35%)	6.94 ± 1.31		20.2 ± 10.6	
Gender					
Male	195 (65%)	5.57 ± 1.68	0.958	14.6 ± 11.6	0.823
Female	105 (35%)	5.77 ± 1.33		15.1 ± 11.1	
Smoking	75 (25%)	5.68 ± 1.57	0.609	15.6 ± 10.6	0.483
DM	120 (40%)	6.08 ± 1.61	**0.004**	14.7 ± 11.6	0.931
HTN	180 (60%)	5.75 ± 1.63	0.892	13.3 ± 10.5	**0.004**
HLP	105 (35%)	6.00 ± 1.48	0.051	14.8 ± 11.8	0.942
BP (mmHg)					
SBP	129.5 ± 29.26	0.038	0.511	0.015	0.801
DBP	79.87 ± 16.82	0.080	0.165	0.037	0.525
EF (%)	30.02 ± 6.686	0.051	0.374	0.033	0.568
Angiography					
Normal *	60 (20%)	4.62 ± 1.29	** < 0.001**	2.25 ± 3.66	** < 0.001**
SVD	48 (16%)	5.50 ± 1.46		12.0 ± 5.81	
2VD	81 (27%)	6.59 ± 1.50		17.6 ± 7.38	
3VD	87 (29%)	6.27 ± 1.28		26.0 ± 8.37	
SF or MB	24 (8%)	4.25 ± 0.67		1.37 ± 2.44	
Management					
Medical	126 (42%)	4.95 ± 1.38	** < 0.001**	4.26 ± 4.68	** < 0.001**
PCI-SV	75 (25%)	6.04 ± 1.38		16.1 ± 4.63	
PCI-MV	30 (10%)	6.80 ± 1.27		21.1 ± 2.00	
CABG	69 (23%)	6.48 ± 1.45		29.9 ± 6.76	
HEART score**	5.760 ± 1.559				
Low	18 (6%)	3.00 ± 0.00		4.66 ± 6.31	** < 0.001**
Moderate	186 (62%)	5.08 ± 0.811	–	12.3 ± 10.7	
High	96 (32%)	7.59 ± 0.789		21.5 ± 10.1	
SS**	14.82 ± 11.38				
Low	225 (75%)	5.52 ± 1.50	** < 0.001**	9.72 ± 7.48	–
Int. and high	75 (25%)	6.48 ± 1.51		30.1 ± 6.13	

*DM* Diabetes mellitus, *HTN* Hypertension, *HLP* Hyperlipidemia, *BP* Blood pressure, *SBP* Systolic pressure, *DBP* Diastolic pressure, *EF* Ejection fraction, *SVD* Single-vessel disease, *2VD* Two-vessel disease, *3VD* Three-vessel disease, *SF* Slow flow, *MB* Muscle bridge, *PCI* Percutaneous coronary intervention, *r* Pearson correlation coefficient, *SD* Standard Deviation, *SV* Single vessel, *MV* Multiple vessels, *CABG* Coronary artery bypass grafting, *SS* SYNTAX score, *Int* Intermediate.

*Mild Coronary Artery disease involvement assumed as Normal.

**Categorization of HEART and SYNTAX scores were done based on the previous definition.

^a^Data of all patients is illustrated by Mean ± SD or Number (%).

^b^Comparison of HEART and SYNTAX scores in different groups were accomplished via One-Way ANOVA and independent T-tests, moreover Pearson correlation coefficient was applied to determine the relationship between these scores and quantitative values.

All statistically significant P values (P < 0.05) are in bold.

In Table 2 all the aspects of the association between the HS and SS are demonstrated. First, we examined quantity values of SS and HS by Pearson correlation and the result was significant (r-coefficient 0.493). Categorization of the SS into low and intermediate/high risk groups and comparison of these sub-groups against HS showed statistically significant differences. One-way ANOVA test confirmed the statistically significant difference in SS between the different HS tertiles as well.

**Table 2:** Different Analyses of association between HEART and SYNTAX scores.

	Pearson correlation coefficient		Independent T-tests	One-way ANOVA
	r-coefficient	P-value	P-value	P-value
Association between quantity values of HEART and SYNTAX scores	0.493	** < 0.001**	–	–
Association between quantity value of HEART score and sub-groups of SYNTAX score	–	–	** < 0.001**	–
Association between quantity value of SYNTAX and sub-groups of HEART score	–	–	–	** < 0.001**

*All statistically significant P values (P < 0.05) are in bold.

The predictive accuracy of the variables derived from different demographic and clinical data was investigated using initially univariate and then multivariate analysis with regard to the SS (Table 3). Age (OR 1.063, 95% CI 1.031–1.096, P < 0.001) and HS (OR 1.282, 95% CI 1.032–1.591, P = 0.025) were independently associated with higher SS. Based on this analysis, for each 1 year increase in age and for each 1 unit increase in HS, the chance of SS being more than 23 will increase by 6% and 28%, respectively. These predictors were analysed by Receiver operating characteristic (ROC) curves to determine their accuracy, sensitivity, and specificity (Table 4).

**Table 3:** Univariate and multivariate analysis of predictors of intermediate and high SYNTAX score (≥ 23).

Variables	Univariate analysis		Multivariate analysis	
	*P*^1^	OR (95% CI)^2^	*P*^1^	OR (95% CI)^2^
Age	** < 0.001**	1.068 (1.040; 1.097)	** < 0.001**	1.063 (1.031; 1.096)
Male gender	0.060	0.599 (0.351; 1.023)	0.481	1.034 (0.422; 1.501)
Smoking	0.817	1.074 (0.584; 1.976)	0.834	1.076 (0.543; 2.129)
Diabetes mellitus	0.415	0.802 (0.473; 1.362)	0.463	0.789 (0.419; 1.486)
Hypertension	0.104	1.549 (0.914; 2.626)	0.096	2.620 (1.303; 5.268)
Hyperlipidemia	0.834	0.943 (0.547; 1.628)	0.429	0.771 (0.404; 1.470)
Systolic blood pressure	0.617	0.998 (0.989; 1.007)	0.109	0.985 (0.967; 1.003)
Diastolic blood pressure	0.282	1.008 (0.993; 1.024)	0.076	1.051 (1.018; 1.085)
Ejection fraction	0.653	1.009 (0.970; 1.049)	0.737	0.992 (0.945; 1.040)
HEART score	** < 0.001**	1.508 (1.259; 1.806)	**0.025**	1.282 (1.032; 1.591)

All statistically significant P values (P < 0.05) are in bold.

^1^Statistical significance of odds ratio.

^2^Odds ratio calculated by univariate and multivariate logistic regression of SYNTAX score and its 95% confidence interval.

**Table 4:** Receiver operating characteristic (ROC) curve analysis for SYNTAX score (≥ 23) prediction.

Intermediate and high SYNTAX score	AUC (95% CI)	Cut-off value	*P* value	Sensitivity (%)	Specificity (%)
Age	0.706 (0.651–0.757)	55 years	< 0.001	88.00	45.33
HEART score	0.671 (0.615–0.724)	6	< 0.001	52.00	74.67

*AUC* Area under the curve, *CI* Confidence interval.

## Discussion

Application of HS, a risk stratification scoring system originally designed for patients presenting with CP^22^, is a non-invasive and practical way of timely approach for these patients at the ED. In the present study, the association between HS and SS, an otherwise invasive risk assessment tool, was assessed. This would help us elucidate the potential of HS for predicting the extension of CAD in CP patients based on SS.

The result of different analyses of the association of HS and SS in the present study revealed that they are indeed positively and significantly related (r = 0.493). This positive relation is further backed up by the result of comparing sub-groups of SS and HS. Furthermore, HS with a cut-off value of 6 is 52% sensitive and 74.67% specific for predicting high SS (≥ 23).

Although studies have already assessed the relation of GRACE and TIMI with SS^23,24^, data regarding our research query is undeniably scarce. Vianna Cedro et al.^25^ in a recent and novel study have demonstrated the association of HEART, GRACE, and TIMI risk scores with angiographic complexity, using SYNTAX, in 138 ACS patients without STE. For the utilization of the ROC curve, they divided patients into two groups of low vs moderate-high SS (≥ 23) and low-moderate versus high SS (> 32). Their results are as follows; a positive and significant correlation was observed between HEART and GRACE score and SS (r = 0.29, P < 0.01 and r = 0.18, P < 0.01 respectively). They found that elevated clinical scores (HEART, GRACE, and TIMI) are predictive of high SS (> 32). Moreover, HS with cut-off points of 5 and 4 were shown to be 64% sensitive and 70% specific and 100% sensitive, and 50% specific for high SS (> 32) respectively. Although their results are in line with ours, some points should be noted. First, their study is conducted on those diagnosed with non-ST ACS whereas we aimed to target a more general population of patients, i.e., CP patients, because HS was primarily developed for risk stratification in CP patients. Second, as discussed earlier, the authors have provided a different cut-off for SS compared to ours and although the presented ROC curve in their study indicates that HS is also predictive of moderate SS, values other than AUC are not presented for SS ≥ 23 in their paper. Nonetheless, the AUC of the HS for moderate SS (≥ 23) is 0.72 (95% CI 0.62–0.83) with P-value < 0.001, which is comparable to our study results.

Additionally, it was shown in our study that HS as well as SS correlated significantly with angiographic results and management, in the sense that lower HS, consequently led to less complex angiographic findings and less aggressive management. Nevertheless, angiographic outcome and management weren't assessed in relation to HS in the above-mentioned study.

As for the remaining results, the aforementioned study by Vianna Cedro et al.^25^ has similarly established a significant relationship between age and SS. Although some conflicts may exist^23^, this relation between age and SS is recognized in other studies as well^24,26^ and our results confirm the same finding. The predictive value of age for SS is considerable to the extent that it is considered as one of the clinical predictors in SYNTAX score II.

In our study, 65% of patients were male and HTN was the main risk factor (60%) followed by DM (40%) and hyperlipidemia (35%). DM was substantially associated with HS, and HTN had a significant association with SS. Vianna-Cedro et al. have also reported that male patients accounted for 68.1% of their population and HTN (83.3%), DM (36.2%), and dyslipidemia (52.2%) were the most common risk factors among them. However, aside from age, they found no significant association between patients’ characteristics and SS. Existing data concerning the association of gender and SS is somewhat contradictory^23,26–28^ and a strong relationship hasn't been established yet. In the same manner, our results have revealed no significant relationship between gender and either of HS and SS. Likewise, even though heart failure and ejection fraction were substantially different in high and low SS in some studies^23,26^, in the present study, no significant association was observed between ejection fraction and either of HS or SS.

Even so, regardless of these discrepancies, demographic and clinical data of patients are so valuable that randomized clinical trials have already developed to incorporate such data into SS. SYNTAX score II, a modified version of SYNTAX based on both anatomical and clinical predictors aiding in more precise and individualized decision making along with the prediction of longer-term mortality in CAD has already been completed^29,30^.

Although our study is multi-centric with a larger sample size compared to previous ones, other clinical scores were not evaluated. Nevertheless, the results of the present study not only emphasize the efficacy of HS, as suggested in previous studies, but also highlight HS as a tool to predict the presence and extension of CAD in CP patients. Despite finding a acceptable association with SS, whether HS can be solely relied on or used along with other risk assessment tools should be investigated in future studies. Moreover, this study was retrospective and the number of participants was relatively low.

## Conclusion

The present study demonstrated that the HEART score has a moderate and positive correlation with the SYNTAX score, highlighting that HEART is potentially a powerful tool for early detection of the extension of CAD in CP patients presenting to the ED. It was revealed that a HEART score of 6 is a predictor for SYNTAX score of ≥ 23.

## Data Availability

The data that support the findings of this study are available from Hamed Bazrafshan Drissi (corresponding author) but restrictions apply to the availability of these data, which were used under license for the current study, and so are not publicly available. Data are however available from the authors upon reasonable request and with permission of Hamed Bazrafshan Drissi.

## Abbreviations

SS: SYNTAX score
HS: HEART score
CP: Chest pain
ACS: Acute coronary syndrome
MACE: Major adverse cardiac events
CAD: Coronary artery disease
ECG: Electrocardiogram
ROC: Receiver operating characteristic
AUC: Area under the curve
PCI: Percutaneous coronary intervention
HTN: Hypertension
DM: Diabetes mellitus
